# Continuous measurement of aortic dimensions in Turner syndrome: a cardiovascular magnetic resonance study

**DOI:** 10.1186/s12968-017-0336-8

**Published:** 2017-02-24

**Authors:** Dhananjay Radhakrishnan Subramaniam, William A. Stoddard, Kristian H. Mortensen, Steffen Ringgaard, Christian Trolle, Claus H. Gravholt, Ephraim J. Gutmark, Goutham Mylavarapu, Philippe F. Backeljauw, Iris Gutmark-Little

**Affiliations:** 1 Department of Aerospace Engineering and Engineering Mechanics, CEAS, University of Cincinnati, Cincinnati, OH USA; 2 Cardio-respiratory Unit, Great Ormond Street Hospital for Children NHS Foundation Trust, London, UK; 3 Institute for Clinical Medicine, Aarhus University, Aarhus N, Denmark; 4 Department of Endocrinology and Internal Medicine, Aarhus University Hospital, Aarhus C, Denmark; 5 Department of Molecular Medicine, Aarhus University Hospital, Aarhus N, Denmark; 6 UC Department of Otolaryngology, Head and Neck Surgery, Cincinnati, OH USA; 7 Division of Pulmonary Medicine, Cincinnati Children’s Hospital Medical Center, Cincinnati, OH USA; 8 Division of Endocrinology, Department of Pediatrics, Cincinnati Children’s Hospital Medical Center, Cincinnati, OH 45229 USA

**Keywords:** Turner syndrome, Aorta, Cardiovascular magnetic resonance, Centerlines, Continuous measures, Maximum diameter, Euclidean distance, Iterative closest point

## Abstract

**Background:**

Severity of thoracic aortic disease in Turner syndrome (TS) patients is currently described through measures of aorta size and geometry at discrete locations. The objective of this study is to develop an improved measurement tool that quantifies changes in size and geometry over time, continuously along the length of the thoracic aorta.

**Methods:**

Cardiovascular magnetic resonance (CMR) scans for 15 TS patients [41 ± 9 years (mean age ± standard deviation (SD))] were acquired over a 10-year period and compared with ten healthy gender and age-matched controls. Three-dimensional aortic geometries were reconstructed, smoothed and clipped, which was followed by identification of centerlines and planes normal to the centerlines. Geometric variables, including maximum diameter and cross-sectional area, were evaluated continuously along the thoracic aorta. Distance maps were computed for TS and compared to the corresponding maps for controls, to highlight any asymmetry and dimensional differences between diseased and normal aortae. Furthermore, a registration scheme was proposed to estimate localized changes in aorta geometry between visits. The estimated maximum diameter from the continuous method was then compared with corresponding manual measurements at 7 discrete locations for each visit and for changes between visits.

**Results:**

Manual measures at the seven positions and the corresponding continuous measurements of maximum diameter for all visits considered, correlated highly (R-value = 0.77, *P* < 0.01). There was good agreement between manual and continuous measurement methods for visit-to-visit changes in maximum diameter. The continuous method was less sensitive to inter-user variability [0.2 ± 2.3 mm (mean difference in diameters ± SD)] and choice of smoothing software [0.3 ± 1.3 mm]. Aortic diameters were larger in TS than controls in the ascending [TS: 13.4 ± 2.1 mm (mean distance ± SD), Controls: 12.6 ± 1 mm] and descending [TS: 10.2 ± 1.3 mm (mean distance ± SD), Controls: 9.5 ± 0.9 mm] thoracic aorta as observed from the distance maps.

**Conclusions:**

An automated methodology is presented that enables rapid and precise three-dimensional measurement of thoracic aortic geometry, which can serve as an improved tool to define disease severity and monitor disease progression.

**Trial registration:**

ClinicalTrials.gov Identifier - NCT01678274. Registered - 08.30.2012.

## Background

Thoracic aortic disease, be it congenital or acquired, is a major determinant of morbidity and mortality in TS [1]. Cardiovascular risk assessment in Turner syndrome (TS), particularly for aortic dissection, unfortunately has remained inadequate, which is due to a limited understanding of the pathophysiology of thoracic aortic disease in TS with aortic events occurring at dimensions classified as normal according to conventional size criteria [2]. Cardiovascular magnetic resonance (CMR) is the gold standard for non-invasive assessment of thoracic aortic disease [3]. CMR has been employed previously in TS, to identify structural aortic anomalies and to follow aortic changes over time [4]. Aortic diameter and growth are the only acknowledged risk markers for aortic dissection [5], and assessment of aortic dimensions using CMR is thereby the established clinical practice for cardiovascular risk assessment in TS [5].

Normative data on aortic dimensions in TS [6] is based on evaluation of aortic diameter at discrete measurement positions, with major determinants including aortic valve morphology, age and blood pressure [7, 8]. A statistical model, based on gold standard CMR, has been developed to assist in the identification of patients with rapid growth in aortic dimensions and to improve clinical decision making [5]. Consistent with international guidelines [9, 10], two-dimensional aortic measurements are performed manually or semi-automatically at discrete locations along the length of the thoracic aorta. Manual measurements are, however, labor-intensive and require multi-plane reformatting to obtain accurate measurements in the correct imaging planes [7], and techniques have been proposed to obtain aorta diameter as well as cross-sectional area automatically in order to overcome some of the challenges in obtaining precise geometric measures using the manual method [11, 12]. Statistical shape modeling methods have been recently developed to quantitatively re-create the three-dimensional (3D) morphology of the aortic arch [13–15] in patients with aortic coarctation and hypoplastic left heart syndrome. In addition, 3D geometric markers employed previously to characterize abdominal aortic aneurysms [16] may further improve the ability to describe thoracic aortic disease in TS.

The present study sets out to devise a novel continuous measurement tool to improve the ability to, in a highly detailed fashion, characterize aortic size and geometry with the aim to improve the ability to diagnose and monitor thoracic aortic disease in TS. To then assess the clinical validity of our novel approach, measurements obtained from the continuous methodology were compared using the same methodology but different algorithms and with the existing manual measurements, and we discuss the preciseness of the approach and present results for the various clinical phenotypes in TS. To further provide insight into thoracic aortic disease in TS, geometric asymmetry and local dimensions of normal and diseased aortae were assessed by introducing a geometric quantity that varies along the aorta length and circumference. To lastly provide basis for future measurement of aortic size in TS a novel registration method was devised to estimate 3D visit-to-visit change in aortic geometry.

## Methods

Fifteen patients, with karyotypically proven TS, were recruited through the Danish National Society of Turner Syndrome contact group and a tertiary endocrine outpatient clinic [5]. Exclusion criteria included malignancy, liver disease, and mechanical aortic valve prosthesis. The TS subjects were examined at baseline (Visit 1) and two subsequent follow-up visits (Visit 2 and 3) over a 10 year period using CMR and transthoracic echocardiography (for aortic valve morphology) [7]. Ten healthy subjects were recruited as baseline controls, and examined once. Table 1 summarizes the age and structural morphologies encountered according to definitions described elsewhere [17, 18].

**Table 1:** Summary of turner syndrome clinical history

Patient	Age (years)	BAV	CoA	ETA
1	47	N	N	N
2	31	N	N	N
3	58	N	N	N
4	57	Y	N	N
5	38	N	N	Y
6	36	Y	N	Y
7	28	Y	Y	Y
8	38	Y	Y	Y
9	45	Y	N	Y
10	39	Y	Y	Y
11	25	Y	Y	Y
12	46	N	Y	N
13	40	N	Y	Y
14	42	N	Y	Y
15	44	N	Y	N

(Note: Patient age corresponds to first visit. *BAV* bicuspid aortic valve, *CoA* coarctation, *ETA* elongated transverse aorta)

### CMR

CMR was performed using a 1.5 T whole-body scanner (Philips Medical System- Best, The Netherlands). A contrast-free, fat-saturated, nearly isotropic, 3D steady-state-free-precession and electrocardiogram (ECG) triggered gradient echo sequence with a respiratory navigator was adopted in this study [5, 8]. The 3D image stack was acquired during the diastolic phase of the cardiac cycle (stack dimensions: 27 cm (anterior-posterior) × 20 cm (feet-head) × 36 cm (left-right)). Spatial resolution was 256 × 256 pixels (pixel spacing: 1.41 × 1.41 mm).

### Manual measurements

Dedicated software (Systematic Software Engineering, Aarhus, Denmark) that allowed multiplanar reformatting of the 3D image stack, was used by two CMR experienced readers to manually measure maximum aortic diameter at eight discrete locations and guided by aortic and extra-aortic landmarks [8]. These locations were: 1) the sinotubular junction 2) the ascending aorta, midway between the sinotubular junction and the innominate artery, 3) the ascending aorta, immediately proximal to innominate artery, 4) the proximal transverse arch, midway between innominate and left carotid artery, 5) the distal transverse arch, just proximal to left subclavian artery, 6) Aortic isthmus, immediately distal to the left subclavian artery, 7) the descending aorta, between left pulmonary artery and top of left atrium, 8) the descending aorta, at the caudal border of the left atrium [7]. For the aforementioned measurement stations, inter-observer variability was: (i) −0.3 (−2.3;1.8) mm; (ii) −0.1 (−1.9;1.4) mm; (iii) 0.1 (−1.6;1.7) mm; (iv) −0.2 (−1.4;1.9) mm; (v) −0.01 (−1.6;1.4) mm; (vi) −0.1 (−1.4;1.9) mm; (vii) 0.08 (−1.1;1.9) mm; and (viii) 0.1 (−1.2;1.5) mm [5]. The corresponding intra-observer measurement variability was: (i) 0.02 (−1.8;1.9) mm; (ii) −0.1 (−1.9;1.8) mm; (iii) −0.1 (−1.9;2.1) mm; (iv) 0.20 (−1.6;2.0) mm; (v) 0.01 (−1.7;1.7) mm; (vi) 0.1 (−1.6;1.4) mm; (vii) 0.08 (−1.5;1.4) mm; and (viii) −0.06 (−1.6;1.7) mm [5].

### Aortic segmentation

**Fig. 1:**
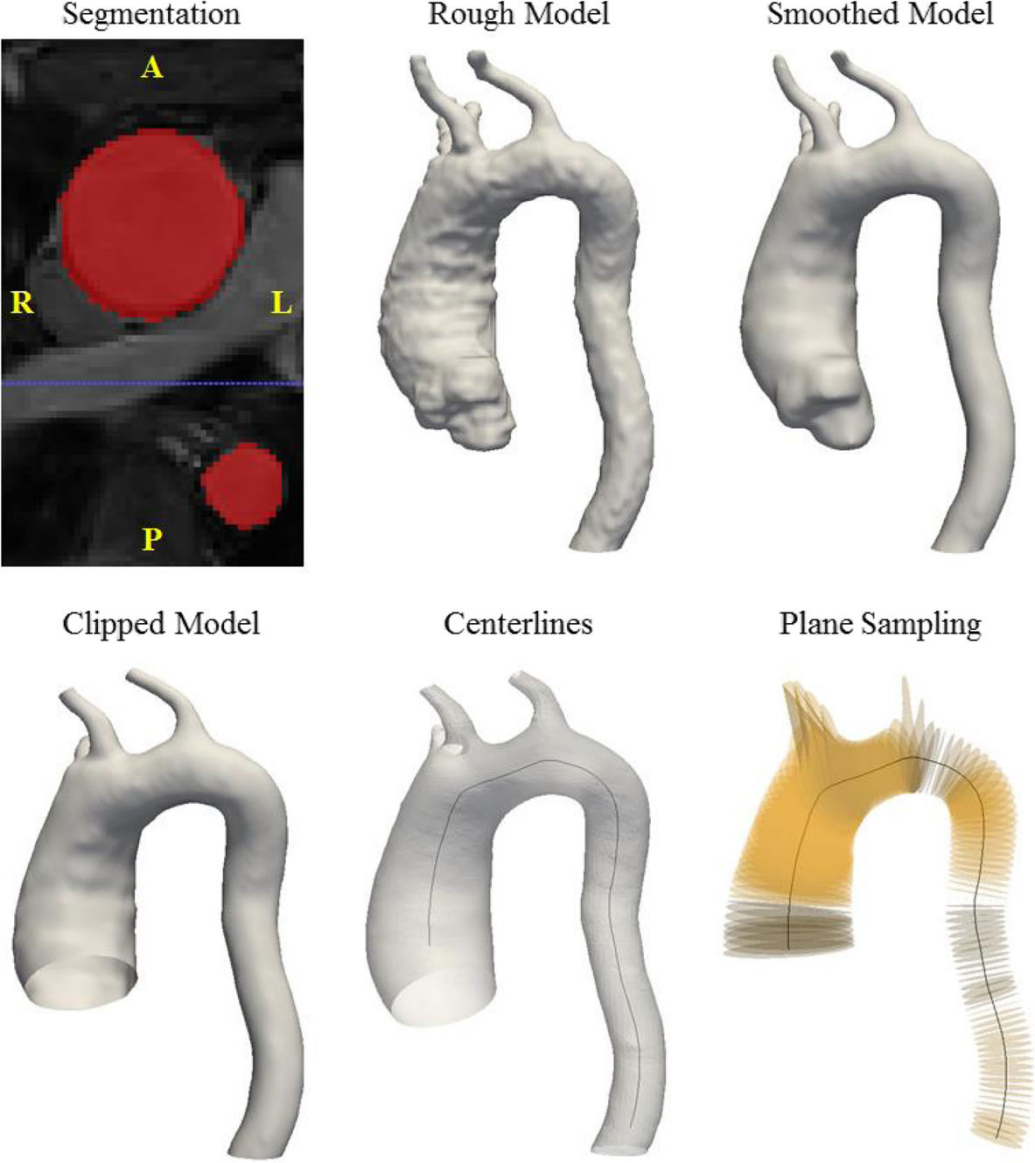
Procedure to generate 3D patient-specific geometries of the thoracic aorta, exemplied for a subject with Turner sydrome (Subject 6). The CMR images were segmented to identify a rough geometry which is subsequently smoothed and clipped for analysis. Centerlines were then identified using VMTK (highlighted in *black*, overlayed on corresponding aorta geometry) and planes could be sampled normal to the centerline (highlighted in *yellow*, centerline also shown for reference)

In order to accurately estimate aorta dimensions without multiplanar reformatting, we first reconstructed the thoracic aortae in 3D. Each CMR data set was then imported into a specialized image-processing software (Mimics, Materialise Inc., Plymouth, MI) to segment the aorta using a thresholding algorithm, as described elsewhere [12] (Fig. 1). Exclusion criteria for segmentation included noisy datasets that exhibited random brightness variations in the aorta lumen and image stacks that did not encompass the entire thoracic aorta (3 out of 45 datasets). The lower and upper threshold values for segmentation of the thoracic aorta ranged from 310 to 640 Grey Values (GV). Geometries of the thoracic aorta were then generated to include the innominate, left common carotid and left subclavian arteries. As shown in Fig. 1, the coarse geometries were smoothed in Mimics to minimize surface artifacts prior to geometric analysis. Smoothed geometries were clipped to identify inlets and outlets of the aorta (Fig. 1) using the Paraview software (Kitware Inc., Clifton Park, NY). The truncated geometries were triangulated using 3-Matic (Materialise Inc., Plymouth, MI) and the lumen surface was exported in stereolithography (STL) format for geometric measurements.

### Centerline extraction and geometric parameter estimation

In order to ensure that maximum diameter measurements were truly perpendicular to the aorta axis [5], we identified the centerline of the reconstructed aortas [12]. The Vascular Modeling Toolkit software (VMTK) [19] has been employed previously to identify centerlines for geometric measurements of the thoracic and abdominal aortae [16, 20]. The open-source version of this software was adopted here. VMTK estimates the centerlines as the weighted, shortest paths traced between two extremal points. The lines are ensured to be central, since they run on the Voronoi diagram (i.e. the location where centers of maximal inscribed spheres are defined) of the vessel geometry. A detailed description of the methodology used to estimate centerlines can be found elsewhere [21]. The centerlines for continuous measurements were identified between the inlet in the ascending aorta and outlet in the descending aorta (i.e. excluding branches) and smoothed using VMTK (Fig. 1). Planes (highlighted in yellow in Fig. 1) spaced 2 mm apart were then sampled normal to the smoothed centerlines using VMTK. Geometric variables including maximum aortic diameter and cross-sectional area were estimated at the individual cross-sections. Centerlines were also employed to calculate tortuosity of the aorta [21] and curvature at the highest point in the aortic arch [12]. The procedure outlined was performed for the 15 TS subjects, including baseline and subsequent follow-up visits, in order to quantify change in aortic dimensions over time. We defined growth or shrinkage as visit-to-visit change in the maximum diameter above inter-observer variability [5]. It should be noted that a sensitivity analysis was performed to test for inter-user variability in aorta diameter measures obtained using the presented approach. We also tested variability in aorta diameter measures with respect to choice of smoothing algorithm, segmentation and smoothing software. The details of this sensitivity analysis are presented in the Appendix.

### Evaluation of localized dimensions and asymmetric change

In order to obtain 3D markers of thoracic aortic morphology, we described methods to estimate localized dimensions and regional changes between visits. We proposed a size field on the aortic surface that was quantified by evaluating the distance from the centerline to each point of the lumen. This geometric variable, known as the Euclidean distance, varies circumferentially (along the vessel periphery) and axially (along the aortic length). This parameter has been employed to quantify diameter growth of the abdominal aorta [16]. We measured the Euclidean distance using VMTK for controls and TS subjects in order to: a) assess the dimensional differences between normal and diseased aorta, and b) quantify the anisotropy (asymmetry) in the aortae dimensions between visits in TS. To measure this variable, a new smoothed centerline was computed for the thoracic aorta and its branches. We also proposed a new method to estimate the asymmetric change between visits (Visit 1 to 2, Visit 2 to 3 and Visit 1 to 3) using an iterative closest point (ICP) registration algorithm, that minimizes the difference between a pair of point clouds [22]. Aortic geometries to be compared were initially registered using the ICP registration available in VMTK [13–15]. The CloudCompare open-source mesh processing software (http://www.cloudcompare.org) was then employed to further align the two aortic surfaces by picking point-pairs. Fine alignment of the aortic surfaces was subsequently achieved by employing the ICP implementation available in CloudCompare. The difference between the aortae (i.e. change between visits) was afterwards assessed using the surface distance module available in VMTK, which computes the minimum point to point distance of the target (i.e. registered) aorta surface (Visit 2 or 3) from the reference (Visit 1 or 2) [23].

### Statistical methods and data analysis

A least-squares linear regression analysis was performed to assess the correlation between manual and continuous measures, Bland-Altman plots were generated to estimate the agreement between these two methods, with the reproducibility co-efficient or limit of agreement computed as ± 1.96SD. In these plots, the difference between the methods was plotted on the ordinate and the average of the continuous and manual techniques was plotted on the abscissa. Horizontal lines were drawn to indicate the mean difference and the upper and lower limits of agreement [24]. In order to test for presence of constant and proportional bias, Passing-Bablok regression analysis was performed. As compared to the least-squares regression method described earlier, the Passing-Bablok regression involves no special assumptions regarding the distribution of samples or measurement methods [25]. These analyses were performed using MATLAB (MathWorks Inc., Natick, MA). An F-test was performed using Microsoft Excel 2010 to estimate the concordance between visit-to-visit change in maximum diameter for the measurement positions, obtained manually and continuously. It should be noted that we compared the *F*-value and corresponding critical value in order to determine equivalence of the variances of change between visits obtained manually and continuously [26]. All continuous variables were indicated as means ± standard deviations. A *p*-value of < 0.05 was considered statistically significant and *R*-values were employed to describe coefficient of correlation.

## Results

### Comparison between methods

**Fig. 2:**
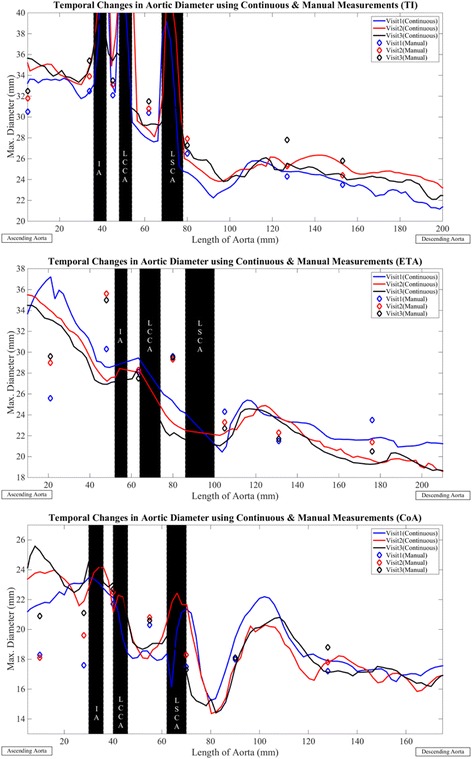
Visit based variation in maximum aortic diameter for three aortic phenotypes in TS comprised of aortic valve regurgitation (Subject 1), elongation of the transverse aortic arch (Subject 9) and aortic coarctation (Subject 7). Visit 1 – *solid blue line*, Visit 2 – solid red line, Visit 3 – *solid black line*. Diamond markers indicate corresponding manual measures. Data at locations of the inominate artery (IA), left common carotid artery (LCCA), left subclavian artery (LSCA) (shaded in *black*) excluded from analysis. All values are in mm. Aortas for the three visits aligned at the branches

Values of maximum aortic diameter and cross-sectional area obtained from continuous measurements were presented as one-dimensional line plots. Geometric measures at the innominate (IA), left common carotid artery (LCCA) and left subclavian artery (LSCA) were excluded from the analysis, with their locations indicated using black bands [20]. Figure 2 indicates three cases, including one subject with regurgitant tri-leaflet aortic valve (Subject 1), a second subject with elongated transverse aorta (ETA) (Subject 9) and a third subject with aortic coarctation (Subject 7), used to demonstrate the ability of the continuous method to quantify visit-by-visit dimensions of the thoracic aorta with manual measures of maximum diameter superimposed. It may be seen, that for instance for the subject with aortic valve regurgitation, maximum diameter predicted using the manual and continuous methods were in concordance throughout the thoracic aorta. Furthermore, aortic diameter measures were consistent over time in the ascending, transverse and descending aorta. For the subject with ETA (Subject 9), both methods depicted reduction in descending aorta diameter with each visit. Descending aorta dimensions were nearly equivalent for the two methods. The continuous method predicted smaller registered diameters in the ascending and transverse aorta. The manual method predicted growth in the ascending segment and stable dimensions in the transverse aorta.

**Fig. 3:**
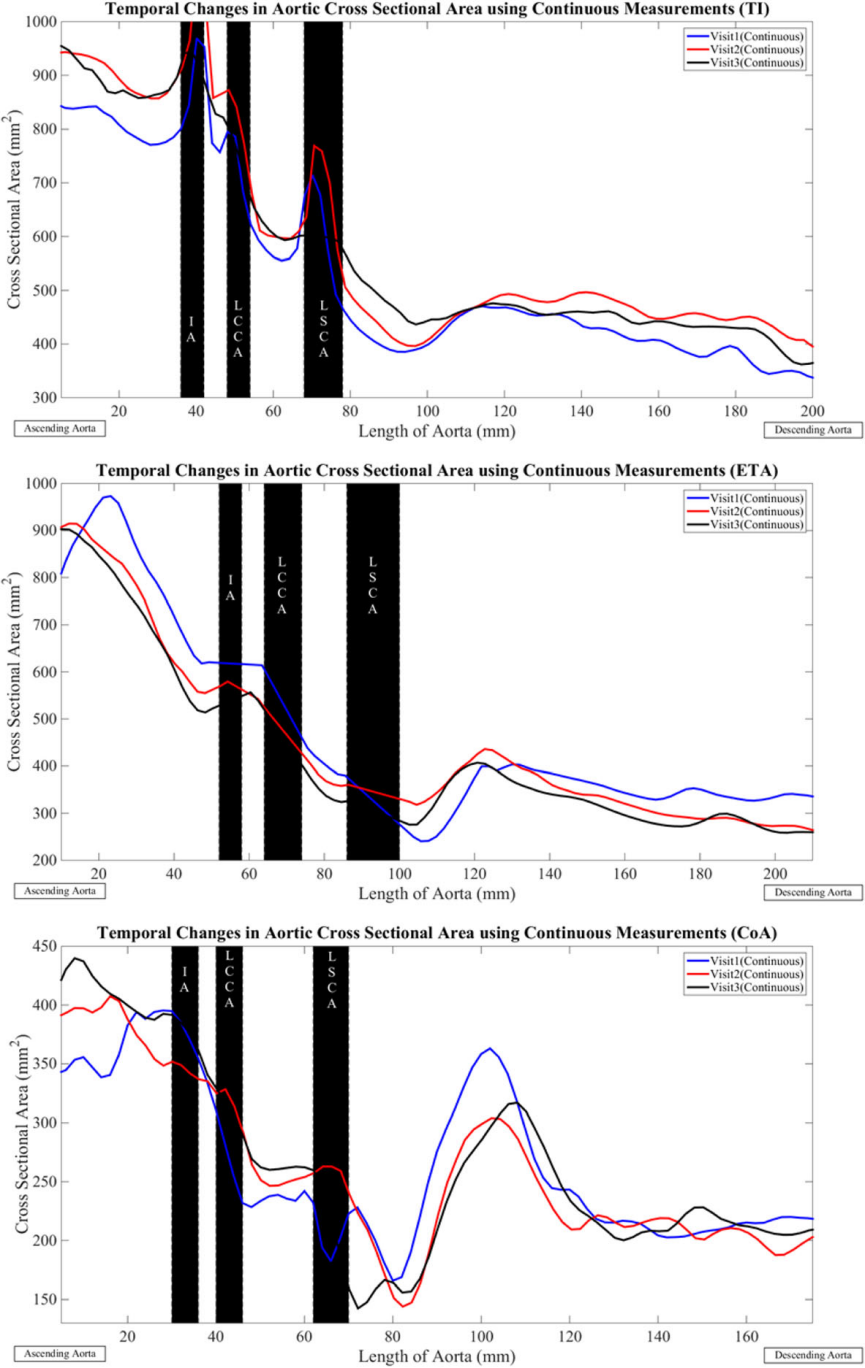
Visit based variation in cross-sectional area for three aortic phenotypes in TS comprised of aortic valve regurgitation (Subject 1), elongation of the transverse aortic arch (Subject 9) and aortic coarctation (Subject7). Visit 1 – *solid blue line*, Visit 2 – *solid red line*, Visit 3 – *solid black line. Diamond markers* indicate corresponding manual measures. Data at locations of the inominate artery (IA), left common carotid artery (LCCA), left subclavian artery (LSCA) (shaded in *black*) excluded from analysis. All values are in mm^2^. Aortas for the three visits aligned at the branches

Both methods predicted growth in the ascending aorta and stable dimensions in the transverse section, for the subject with aortic coarctation (Subject 7) (Fig. 2). The continuous method predicted smaller registered diameters in the descending aorta and the manual method indicated stable dimensions over time. The continuous method predicted larger values of maximum diameter in the ascending aorta for all visits considered in this study. Maximum diameter values obtained from the two methods were similar in sections of the transverse and descending aorta. The coarctation and its location (immediately following the LSCA), was expressed more accurately by the continuous method. The visit-by-visit variation in lumen cross-sectional area for the three subjects is indicated in Fig. 3. As can be seen, the trends for variation in area were nor surprisingly similar to the variation in maximum diameter (Fig. 2). For TS, curvature at the highest point in the aortic arch was greater for all visits (Visit 1: 0.3 ± 0.2 (1/mm), Visit 2: 0.6 ± 1.1 (1/mm), Visit 3: 0.4 ± 0.3 (1/mm)) as compared to controls (0.2 ± 0.1 (1/mm)). Similar trends in aortic arch curvatures were observed between patients with post-coarctation repair, patients with post-arterial switch operation and healthy subjects [12]. Aorta tortuosity was also lower for the healthy individuals (1.3 ± 0.2) as compared to the TS patients for all visits considered in our study (Visit 1 and 3: 1.5 ± 0.3, Visit 2: 1.6 ± 0.3).

**Fig. 4:**
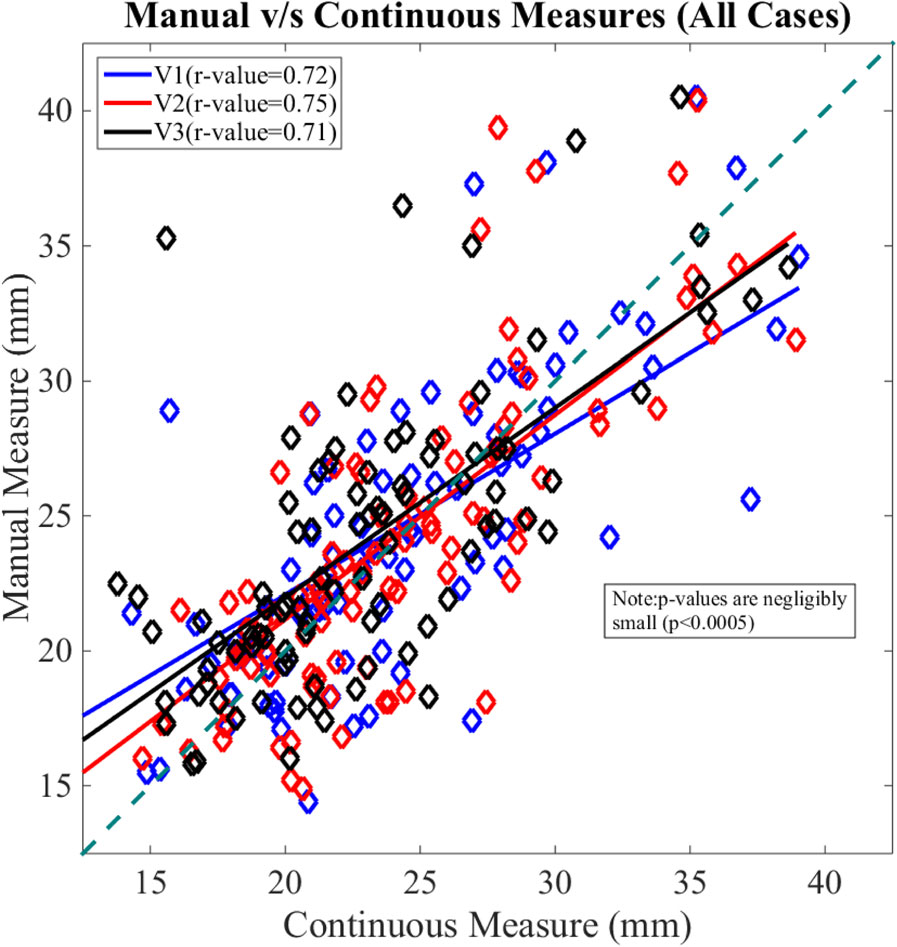
Scatter plot and linear regression lines for maximum diameter at select locations along the thoracic aorta for all cases and visits. Visit 1 - *blue diamond markers* and *solid line*, Visit 2 - *red diamond markers* and *solid line*, Visit 3 - *black diamond markers* and *solid line*. Forty-five degrees *dashed green line* also shown to indicate deviation of manual measures relative to the corresponding continuous values

**Fig. 5:**
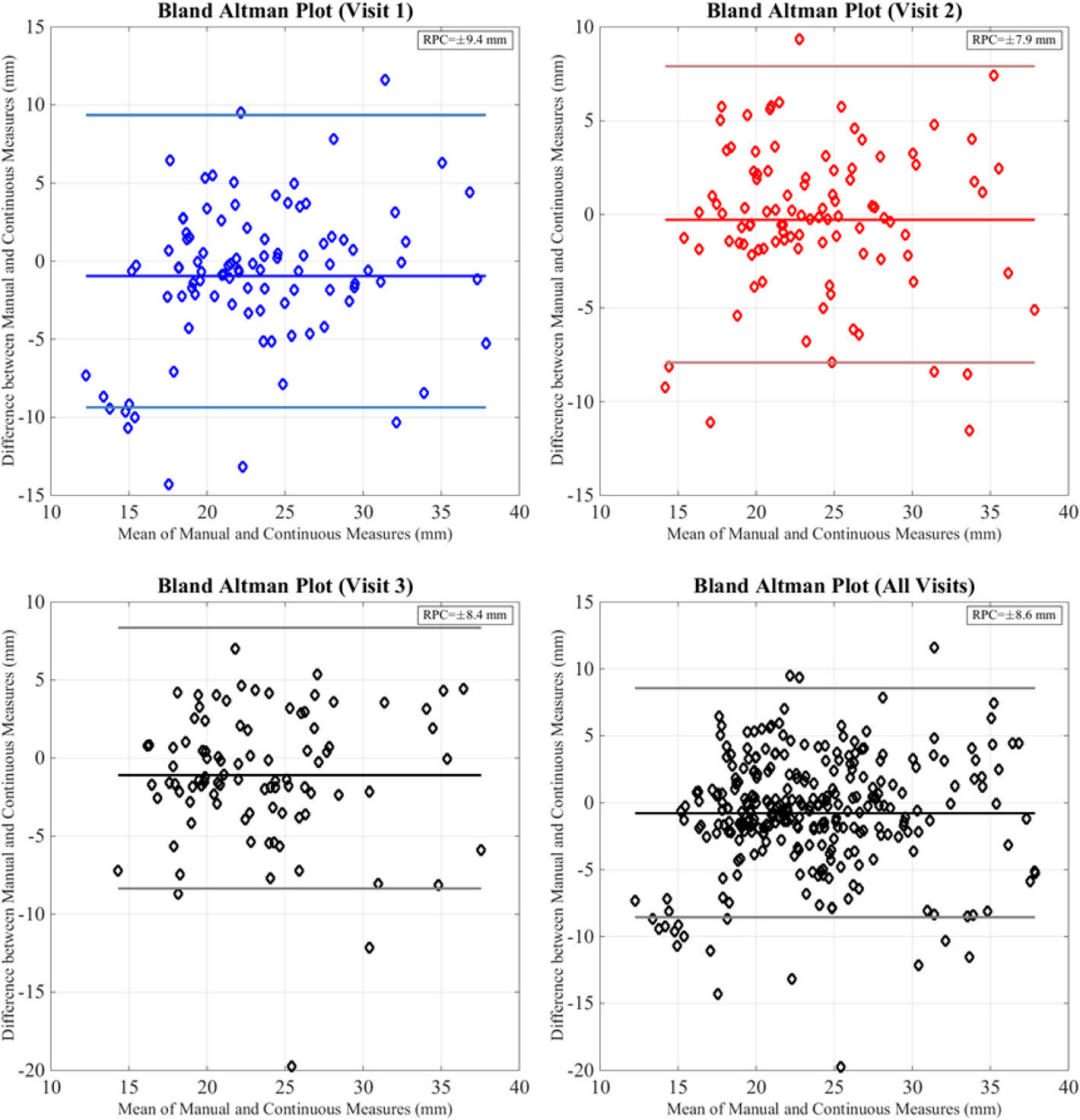
Bland-Altman analysis comparing maximum aortic diameter obtained using manual and continuous methods for individual visits and all visits considered. Visit 1 - *blue* diamond markers, *solid blue line* – mean, *light blue lines* - ±1.96SD, Visit 2 – *red diamond markers*, *solid red line* – mean, *light red lines* - ±1.96SD, Visit 3, All Visits - *black diamond markers*, *solid black line* – mean, *gray lines* - ±1.96SD

**Fig. 6:**
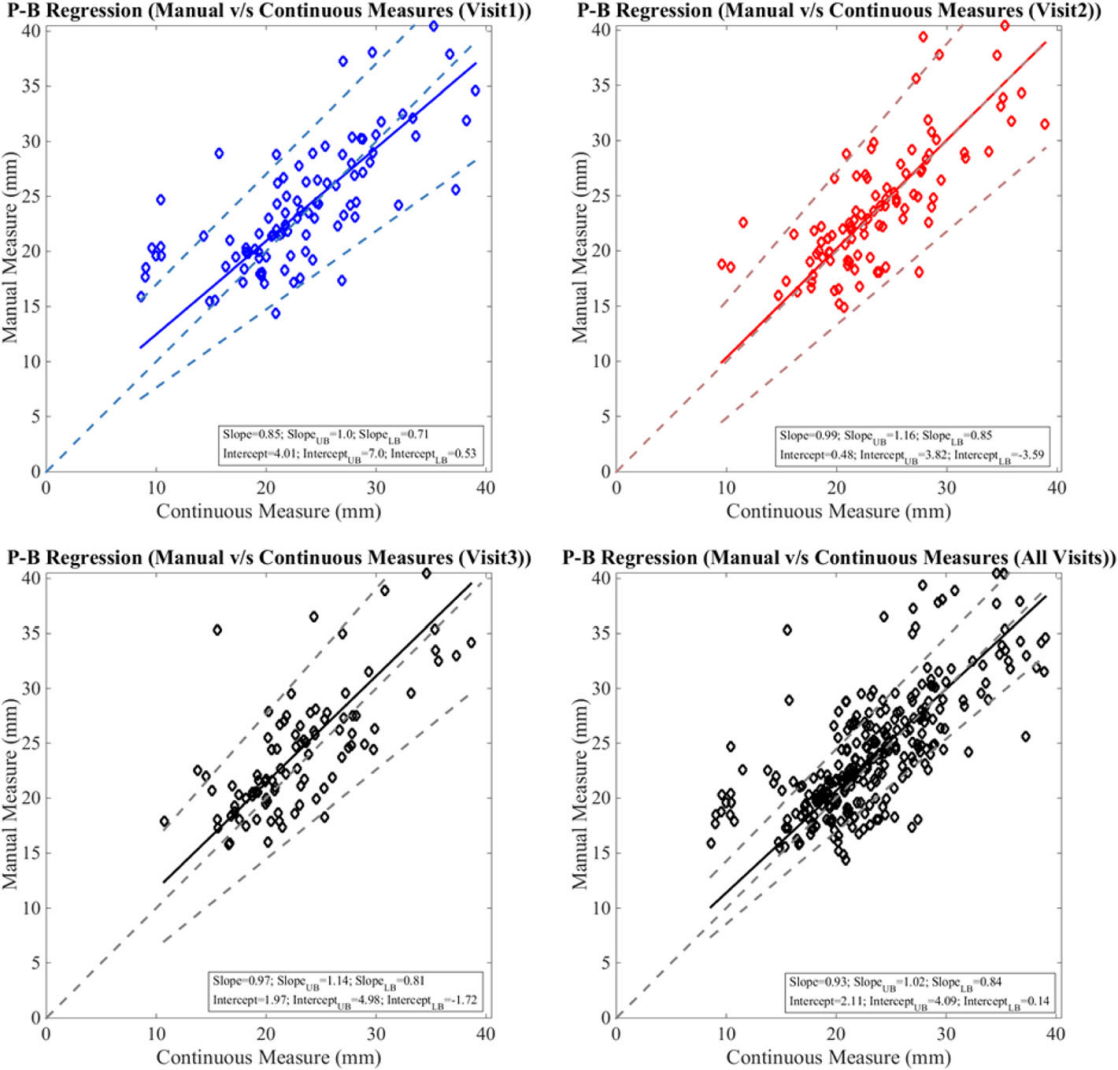
Passing-Bablok regression plots comparing manual and continuous methods for individual visits and all visits considered. Visit 1 - *blue diamond markers*, *solid blue line* – regression line, light *blue dashed lines* – upper and lower bounds, Visit 2 – *red diamond markers*, *solid red line* – regression line, *light red lines* – upper and lower bounds, Visit 3, All Visits - *black diamond markers*, *solid black line* – regression line, *gray lines* – upper and lower bounds

Scatter plots and least-squares based linear regression lines comparing maximum diameter obtained manually and continuously, at the discrete measurement positions, for all cases and visits are indicated in Fig. 4. The overall least-squares regression coefficient was 0.77 (*p*-value < 0.01) and was equivalent to the correlation coefficient reported in a previous study [11]. Similar correlation coefficients were obtained for the individual visits (Visit 1: R-value = 0.72, Visit 2: R-value = 0.75, Visit 3: R-value = 0.71, all *P* < 0.005). The Bland-Altman plots (Fig. 5) showed a slight negative bias of −0.78 mm between the manual and continuous methods for all visits considered together. The reproducibility coefficient was 8.6 mm. For the baseline (Visit 1) and second follow-up (Visit 3) visits, a negative bias was observed between the two methods (Visit 1:-0.96 mm, Visit 3:-1.1 mm) as compared to Visit 2 (−0.29 mm). The corresponding limits of agreement were also smaller for Visit 2 (±7.9 mm) compared to Visit 1 (±9.4 mm) and Visit 3 (±8.4 mm). Analysis of the Passing-Bablok regression (Fig. 6) parameters indicated significant constant bias between the manual and continuous methods overall and for the individual visits considered in our study (Table 2). The bias was least significant for the first follow-up (Visit 2) and greatest for the baseline visit (Visit 1). In addition, the slopes were lower than one and indicated the presence of proportional bias between the two methods. The proportional bias was observed to be most significant for aorta maximum diameter measurements at Visit 1. Table 3 summarizes the F-test correlations for different sections of the thoracic aorta. As can be seen, the visit-by-visit changes for the complete thoracic aorta and the individual segments correlated well (F < F_crit_).

**Table 2:** Passing-Bablok regression analysis comparing maximum aortic diameters obtained using manual and continuous methods (Note: Values in parenthesis indicate lower and upper bounds)

Visit	Intercept (95% CI)	Slope (95% CI)
1	4.01 (0.53 to 7.0)	0.85 (0.71 to 1)
2	0.48 (−3.59 to 3.82)	0.99 (0.85 to 1.16)
3	1.97 (−1.72 to 4.98)	0.97 (0.81 to 1.14)
All Visits	2.11 (0.14 to 4.09)	0.93 (0.84 to 1.02)

**Table 3:** Summary of correlations of change between visits obtained using continuous and manual measurement methods (Note: F-test employed in all cases. F-value and corresponding critical value were compared in order to determine equivalence of the variances of change between visits obtained manually and continuously. For good concordance between methods we tested whether F < F_crit_ [26])

Case	F < Fcrit
Overall Visit 1 to 2	Y
Overall Visit 2 to 3	Y
Overall Visit 1 to 3	Y
Ascending Visit 1 to 2	Y
Ascending Visit 2 to 3	Y
Ascending Visit 1 to 3	Y
Transverse Visit 1 to 2	Y
Transverse Visit 2 to 3	N
Transverse Visit 1 to 3	Y
Descending Visit 1 to 2	N
Descending Visit 2 to 3	Y
Descending Visit 1 to 3	Y

### Variation in localized change

**Fig. 7:**
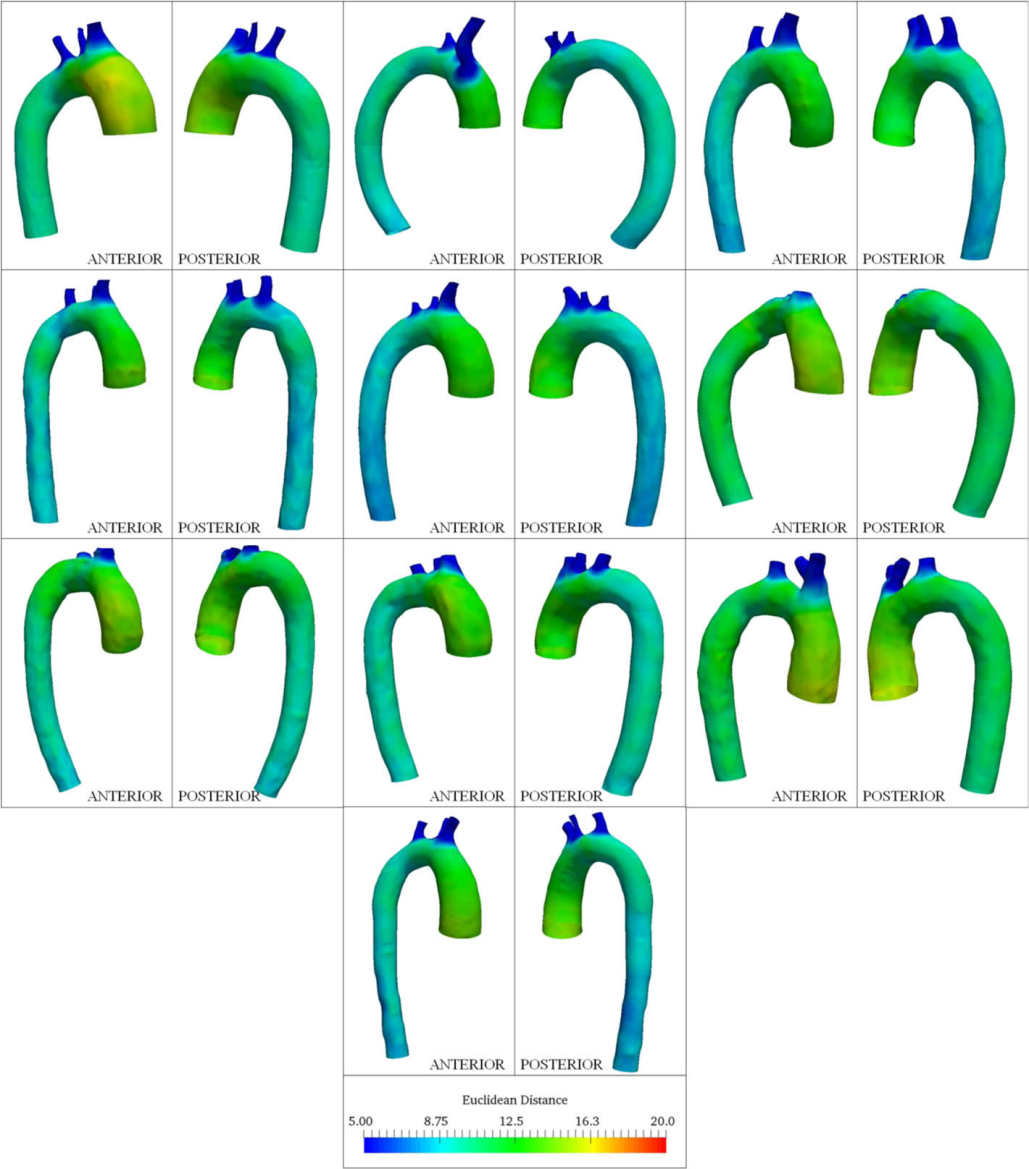
Color plots indicating circumferential and axial variation in Euclidean distance from centerlines for controls included in this study. All dimensions are in mm. Anterior and posterior views of the aorta are shown for each case

**Fig. 8:**
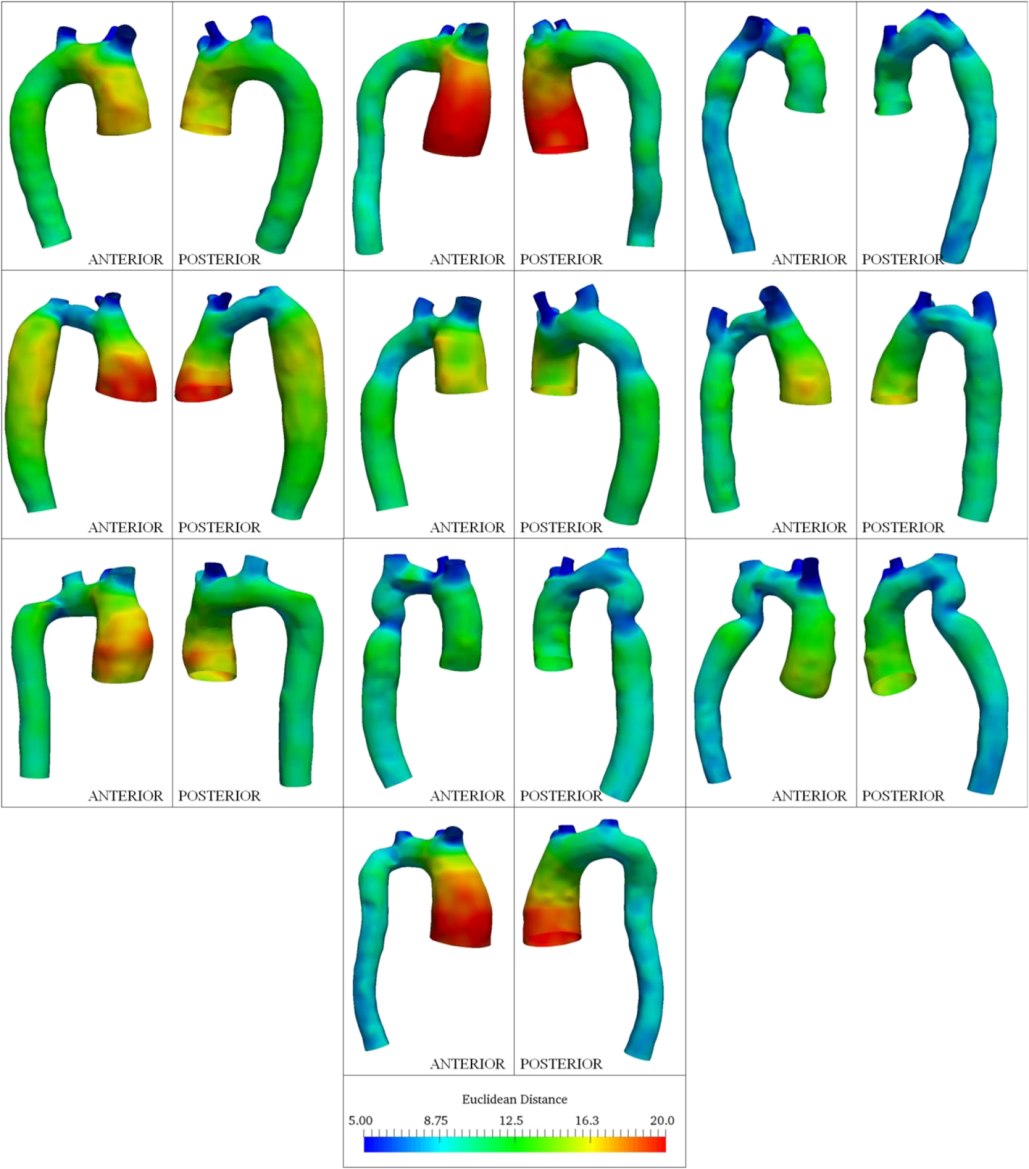
Color plots indicating circumferential and axial variation in Euclidean distance from centerlines for 10 TS patients (patient nos. 1, 4, 5, 6, 8, 9, 10, 11, 13 and 15). All dimensions are in mm. Anterior and posterior views of the aorta are shown for each subject

Among the ten controls, the ascending aorta was larger (12.6 ± 1 mm (mean Euclidean distance ± standard deviation)) than the descending aorta (9.5 ± 0.9 mm) and transverse aortic arch (Fig. 7). Likewise for the TS subjects, the ascending aorta was larger (13.4 ± 2.1 mm) than the descending segment (10.2 ± 1.3 mm). Figure 8 summarizes distance maps for ten subjects (baseline visit: case no. 1, 4, 5, 6, 8, 9, 10, 11, 13 and 15), who represent the various aorta phenotypes in TS. The maximum Euclidean distance in the ascending aorta for TS, averaged over all visits, was greater than the corresponding values for controls (F > F_crit_), and similar for the descending aorta segments (F < F_crit_). Greater asymmetry (anisotropy) in the Euclidean distance was observed throughout the thoracic aorta in TS compared to controls. It should be noted that for a circular (i.e. symmetric) cross-section, the color (value of Euclidean distance) would be unchanged along the aortic circumference. The visit-by-visit Euclidean distance was nearly unchanged throughout the aorta. Table 4 summarizes the maximum and mean distances for the ascending and descending aorta.

**Table 4:** Summary of Euclidean distance variation (mean ± standard deviation, mm) for the ascending and descending thoracic aorta in controls and TS

	Euclidean distance	Controls	TS (Overall)	TS (V1)	TS (V2)	TS (V3)
ASCENDING	*Mean*	12.6 ± 1	13.4 ± 2.1	13.4 ± 2	13.4 ± 2.2	13.3 ± 2.3
	*Maximum*	14.8 ± 0.95	16.5 ± 2.7	16.8 ± 2.8	16.6 ± 2.8	16.1 ± 2.6
DESCENDING	*Mean*	9.5 ± 0.9	10.2 ± 1.3	10.3 ± 1.4	10.3 ± 1.4	10 ± 1.1
	*Maximum*	11.9 ± 1	13.2 ± 1.6	13.2 ± 1.5	13.4 ± 1.8	13 ± 1.5

**Fig. 9:**
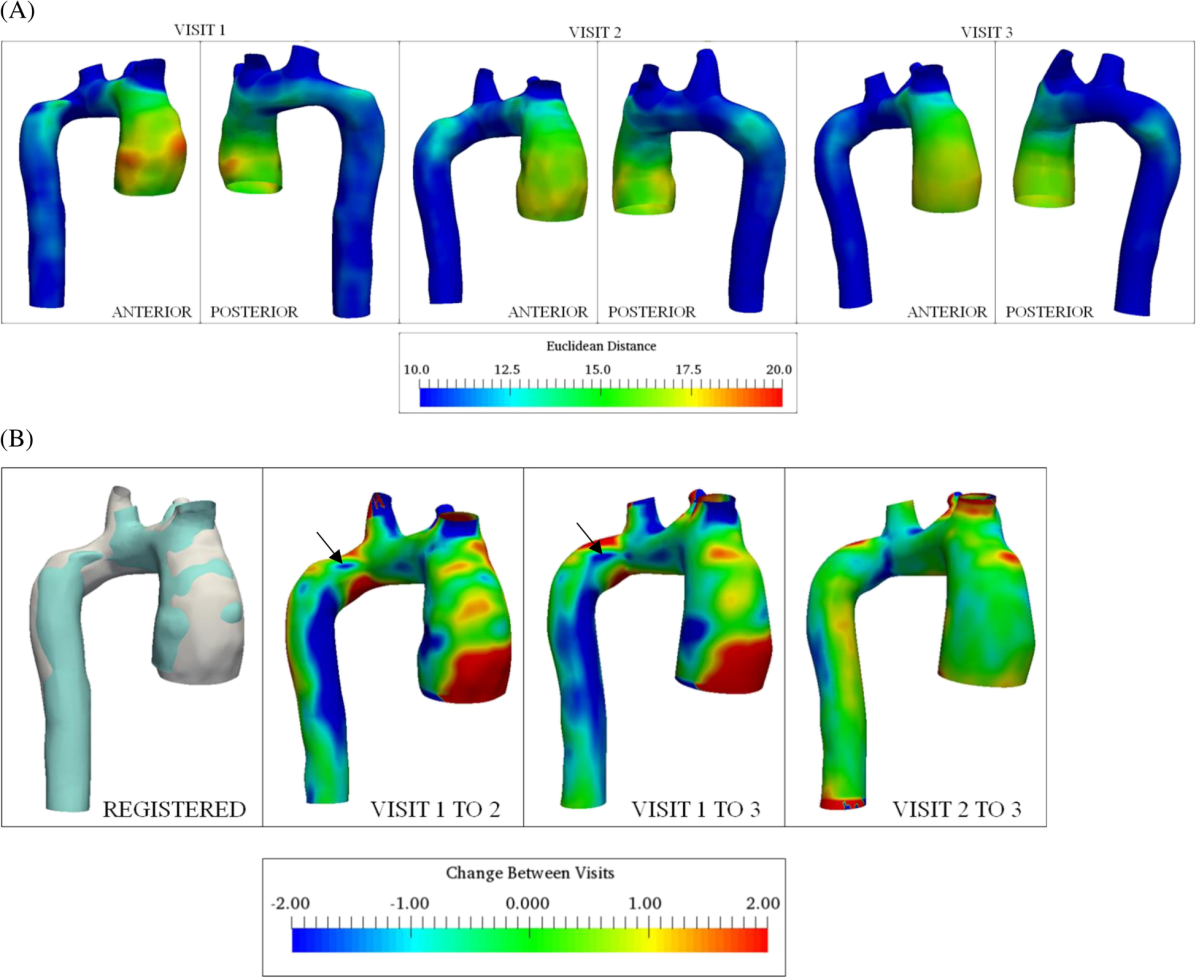
Three dimensional visit-to-visit variation in aorta geometry (Subject 9). **a** Color plots indicating circumferential and axial variation in Euclidean distance from centerlines. All dimensions are in mm. Anterior and posterior views of the aorta are shown for each visit. **b** Color plots indicating circumferential and axial variation in visit-by-visit change, obtained using point registration. Positive indicates increasing and negative indicates decreasing dimension of Visit 2 / Visit 3 relative to Visit 1 / Visit 2. Reference aortic surface (Visit 1 or 2) and registered aorta (Visit 2 or 3) are shown in *blue* and *white*, respectively. All values in mm. Anterior views shown for the three cases. Arrows shown to indicate location of progressive coarctation

The visit-by-visit asymmetry in the aorta cross-section is illustrated for Subject 9 in Fig. 9a. The anisotropy in the ascending aorta (proximal to the aortic root) was larger for Visit 1 compared to Visit 2 and 3, as observed in the anterior view. At the aortic isthmus distal to the LSCA, the out-of-circularity was similar for Visit 1 and 2 and more pronounced than Visit 3. The 3D change in aorta geometry between visits for patient 9, obtained using the iterative closest point registration is presented in Fig. 9b. The reference surface indicated in blue (Visit 1 or 2) and target surface to be compared (either Visit 2 or 3) indicated in white are shown for clarity; positive and negative values implies growth and decrease, respectively. As shown in Fig. 9b, growth resulted anteriorly from Visit 1 to 2 and 1 to 3 in the ascending aorta and in the descending aorta. Apparently progressive aortic coarctation (CoA) was observed to occur in the follow-up visits anteriorly in the descending aorta, downstream of position 7. The 3D plots of geometry change and Euclidean distance maps for this subject correlated well with the corresponding line plots of variation in maximum diameter indicated in Fig. 2.

## Discussion

We present a novel continuous method for assessment of thoracic aortic diameter and geometry. The continuous method has several advantages over the manual approach. Firstly, the continuous method eliminates the need for measurement at both identical locations and cross-sectional angles. Secondly, the continuous measurement method provides complete anatomical information about the entire thoracic aorta. Thereby, the new method is capable of highlighting features, such as the extent and location of any aortic abnormalities and localized increment or decrement, including out-of-circularity changes, unlike manual single-point measurements. It also produces optimum measurements near steep gradients (i.e. at CoA or near branch points), where manual overestimation may occur. Least-squares and Passing-Bablok regression analysis indicated good concordance between values of maximum diameter at the discrete measurement positions obtained using manual and continuous methods. The continuous method was observed to generally predict a higher diameter at different stations along the aorta as indicated by slope values less than one for the least-squares and Passing-Bablok regression lines. The negative bias obtained using the Bland-Altman plots also provided evidence of this trend. Differences in positioning of the discrete measurement stations for the manual or continuous measurement methodology would potentially increase the bias obtained from the Passing-Bablok regression analysis. The outliers indicated in the Bland-Altman plots (Fig. 5) corresponded to aorta scans that concluded at station 8 (i.e. at the caudal border of the left atrium). Improved concordance between the methods could be potentially obtained by extending the scans below the diaphragm level (i.e. inclusion of the abdominal aorta). In addition to the statistical approach presented in this study, validation of aorta lumen segmentation would have to be performed using probabilistic models to minimize under or over-estimation of aortic diameter and enhance the agreement between the continuous and manual methods. The same can be achieved by considering a set of segmentations of the aorta lumen performed manually and automatically and computing a probabilistic estimate of the true segmentation and performance level represented by each segmentation [27]. The presented method estimated stable aortic dimensions over time for most cases as compared to the manual method, which predicted larger changes. The need for unnecessary intervention based on overestimation of growth may thus be potentially minimized with the continuous approach. This approach prevents missing changes between manual locations and neighboring points and establishes continuity and validation. A previously proposed automated method involved fitting of an elliptic cross-section to identify the minimum and maximum aorta diameters [11]. Our method could potentially provide a better estimate of the true maximum diameter given the full circumferential cross-sectional information. Moreover, the plots describing the variation in cross-sectional area (Fig. 3) could be used to obtain measures of localized aortic compliance and the corresponding change over time [12]. The Euclidean distance maps enabled us to not only elucidate dimensional differences between TS and controls, but also to recognize regions that exhibited the greatest asymmetry, and may also provide a better understanding of the aortic pathophysiology in Turner syndrome, especially the localized changes that seem to take place over time. The presented approach to estimate 3D growth or shrinkage is a multi-step registration process that requires low computational effort. Statistical shape atlas techniques based on deformable registration have been demonstrated to precisely reconstruct complex aorta shapes for several cardiovascular disorders [14, 15], by employing suitable values of transformation resolution and stiffness parameters to capture small features [13]. These computational template methods would be considered in a future study to quantify visit-to-visit change in aorta morphology for TS patients.

The continuous measurement method was observed to be more sensitive to the choice of segmentation software and smoothing algorithm as compared to the inter-user variability or choice of smoothing software. Recognizing this large variability, surface area measurements using the different segmentation software were compared with the corresponding analytical values for a modified 3D Shepp-Logan phantom. The errors in surface area measurements were: a) Mimics: 0.8 ± 4.6%, b) ITK-Snap: 1.5 ± 4.5%, c) 3D Slicer: 1.1 ± 4.7%. The absolute error in surface area values was greater for 3D Slicer (~1.5%) as compared to Mimics (~0.2%) and ITK-Snap (~0.4%), for ellipsoids with gray values similar to that of the aorta. Although these aforementioned observations imply that Mimics is potentially more suitable for reconstruction of the aorta geometry using CMR, further validation of the same would be performed in a future study using cardiac phantoms [28]. The average inter-user variability was evaluated to be approximately one pixel (~1.5 mm) and was similar to the values reported previously [5]. Errors in measurement of the aorta diameter using the presented method stem from a variety of sources. Firstly, segmentation of the thoracic aorta was achieved by setting the upper and lower threshold values. Variation in the threshold could potentially result in over or under prediction of the aorta lumen, prior to surface smoothing. Noisy images and low spatial resolution significantly influence the choice of threshold levels needed to segment the vessel lumen. Secondly, smoothing of the aortic geometries was achieved using a fixed number of iterations and smoothing factor. Few iterations or a low smoothing factor could result in more surface artifacts. Smoothing of the centerline prior to sampling of planes could lead to variations in the reference point needed to evaluate the maximum aortic diameter. Thirdly, visit-to-visit changes in the orientation of the branch arteries from the transverse arch present challenges in accurately aligning the aortae necessary to compare geometry changes over time, especially in the transverse section. Correlation with manual measurements is also subsequently influenced by the preciseness of this alignment. Our study assumes no variations in the ECG triggering between visits (i.e., all scans are acquired during the same time point in the diastolic phase of the cardiac cycle). The TS subjects examined were prescribed anti-hypertensive treatment during the first follow-up visit. Changes in diastolic pressure influenced by the treatment and variations in hemodynamic flow variables such as wall shear stress with time [29] could potentially alter the aortic geometry between visits. Additionally, increasing aortic stiffness with age [30] could generate changes in aortic morphology for a fixed aortic pressure. Dilatation of the aortic root may be observed in TS [31, 32]. The methods presented here have been enhanced to include aorta sinuses for 3D measurements of aorta morphology in controls and TS, the details of which would be presented in a future study. The methodology and descriptive statistics reported in this study were performed for a relatively small sample size of 15 subjects, and future studies would include additional diseased subjects and longitudinal scans for healthy individuals. However, with the present sample size we are able to demonstrate the validity of our novel approach. Hierarchical clustering has been employed previously to detect patterns of aortic dilatation in patients with BAV disease [33]. A similar cluster analysis will be performed in a future study to define growth or shrinkage patterns of the aorta in TS.

Evaluating hemodynamic parameters and aortic flow patterns in patient-specific deformed aortae is one of the further necessary steps to improve risk stratification of aortic disease in TS [34, 35]. The one-dimensional continuous measurement method proposed in this study has been previously utilized to explain changes in aortic flow patterns over time, in animal models [20] and the same approach may prove valuable in a future research into associations of variation in flow patterns and wall shear stress with changes in aortic geometry in TS. Spiraling nature of blood flow in the aortic arch has also been observed to correlate with aortic arch curvature [36] and tortuosity [37]. Furthermore, correspondence between mechanical stresses and Euclidean distance maps has been reported previously for dilated aortae [38, 39]. In addition to the statistical model based on manual measurements developed previously by us [5], a mathematical model based on continuum mechanics has also been described previously to predict vessel growth [40, 41]. The changes between visits obtained using one-dimensional line plots and 3D surface change maps could be utilized to validate and advance models of growth prediction and disease progression such as the aforementioned. Surgical planning for interventions on diseased thoracic aortae or aortic valves [42] requires precise dimensional information prior to surgical intervention [11]. The measurement approaches proposed in the study can be used for such pre-operative surgical planning. The described methodology improved to include the aortic sinus, can be applied to cardiovascular disorders such as Marfan syndrome and comparative studies between conditions such as TS and other aortopathies would potentially be of value in delineating differences in pathophysiology and how this manifests in different geometries and changes over time.

## Conclusions

A novel automated continuous measurement tool of maximum aortic diameters was presented in TS, which offers several advantages including: a) elimination of individual user bias resulting in more accurate and reproducible monitoring of abnormal changes in aorta geometry; b) robustness of data and assessment of the entire aortic geometry (i.e. maximum diameter and circumferential location, area, aspect ratio, curvature, tortuosity); c) significant decrease of time spent when manually obtaining aortic dimensions following segmentation of the aorta (less than 5 min per visit), reducing costs. Furthermore, a new 3D method to quantify aortic anisotropy was devised that may improve representation of aortic morphology and could potentially enable better identification and characterization of additional subgroups of aortic phenotypes based on cardiovascular disease progression over time.

## Figures and Tables

**Fig. 10:**
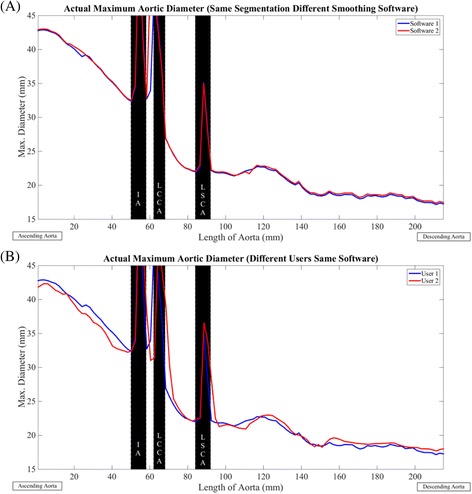
Sensitivity of continuous measurements to choice of segmentation software and smoothing algorithm, exemplified for a subject with Turner syndrome (Subject 6). **a** Software 1 (*solid blue line*) – Mimics, Software 2 (*solid red line*) – ITK-Snap, Software 3 (*solid black line*) – 3D Slicer. **b** Smoothing Algorithm 1 (*solid blue line*) – C0 Smoothing, Smoothing Algorithm 2 (*solid red line*) – C1 Smoothing, Smoothing Algorithm 3 (*solid black line*) – Simple Smoothing. Aorta geometries were smoothed using OpenFlipper

**Fig. 11:**
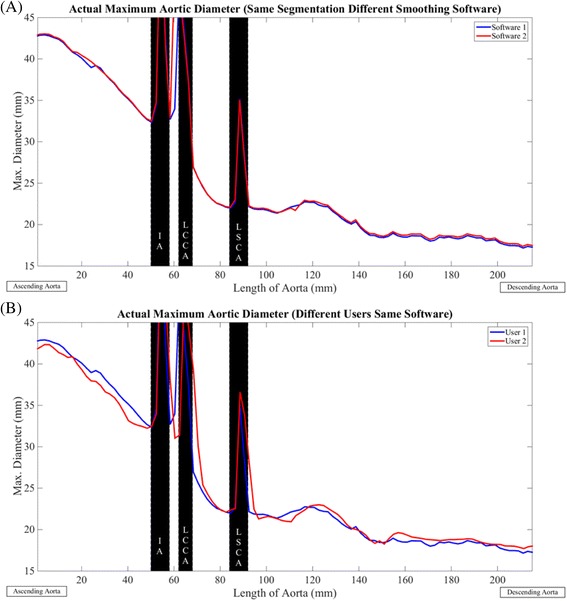
Sensitivity of continuous measurements to choice of smoothing software and user, exemplified for a patient with Turner syndrome (Subject 6). **a** Segmentation of aorta performed using Mimics. Smoothing Software 1 (*solid blue line*) – Mimics, Smoothing Software 2 (*solid red line*) – OpenFlipper. **b** *Solid blue line* – User 1, *Solid red line* – User 2

## Appendix

In order to evaluate the sensitivity of the continuous measurement methodology to the choice of segmentation software, we also tested the methodology using both ITK-Snap [43] and 3D Slicer software (http://www.slicer.org) for identification of the aorta. The same threshold values were used for the three different approaches to aortic segmentation. We also tested the simple, C0 and C1 smoothing algorithms available in the OpenFlipper software [44], to assess the sensitivity of measurements to the choice of smoothing software and smoothing methodology (Figs. 10 and 11). The simple or Laplacian smoothing method adjusts the location of each vertex to the geometric center of its neighboring vertices [45]. C0 smoothing assumes the individual curves defining the surface to remain connected at common vertices and C1 smoothing ensures tangential continuity between the curves [46]. In order to evaluate inter-user variability in the measurements obtained continuously, we compared aorta geometries reconstructed and smoothed by two users independently using Mimics (Fig. 11).

Figure 10 depicts the sensitivity of continuous measurements to the choice of segmentation software and smoothing algorithm in a sample subject (Subject 6), and when comparing different segmentation software with identical smoothing, the maximum diameter measures obtained using Mimics and ITK-Snap were in good agreement compared to 3D Slicer (Difference between Mimics and ITK-Snap: −0.01 ± 2.5 mm, Difference between Mimics and 3D Slicer: −1.4 ± 3.6 mm, Difference between ITK-Snap and 3D Slicer: −1.4 ± 3.5 mm). Regarding the impact of using different smoothing algorithms exemplified for the same subject (Subject 6) in Fig. 10, the diameters obtained using C0 and simple smoothing were comparable. The C1 smoothing resulted in a coarser lumen surface as compared to the C0 or simple smoothing (Difference between C0 and simple: 0.4 ± 0.6 mm, Difference between C0 and C1: 1 ± 1.5 mm, Difference between simple and C1: −0.5 ± 1.2 mm). Figure 11 indicates the sensitivity of continuous measures to choice of smoothing software and inter-user variability in the same subject (Subject 6). The inter-user variability in maximum diameter was 0.2 ± 2.3 mm, and the differences in measures resulting from selecting the simple smoothing available in Mimics or OpenFlipper was 0.3 ± 1.3 mm.

## Abbreviations

3D: Three-dimensional
BAV: Bicuspid aortic valve
CMR: Cardiovascular magnetic resonance
CoA: Coarctation
ECG: Electrocardiogram
ETA: Elongated transverse aorta
GV: Grey values
IA: Innominate artery
ICP: Iterative closest point
LCCA: Left common carotid artery
LSCA: Left subclavian artery
STL: Stereolithography
TI: Regurgitant tri-leaflet aortic valve
TS: Turner syndrome
VMTK: Vascular modeling toolkit
